# Placental Sequestration of *Plasmodium falciparum* Malaria Parasites Is Mediated by the Interaction Between VAR2CSA and Chondroitin Sulfate A on Syndecan-1

**DOI:** 10.1371/journal.ppat.1005831

**Published:** 2016-08-24

**Authors:** Marina Ayres Pereira, Thomas Mandel Clausen, Caroline Pehrson, Yang Mao, Mafalda Resende, Mads Daugaard, Anders Riis Kristensen, Charlotte Spliid, Line Mathiesen, Lisbeth E. Knudsen, Peter Damm, Thor G. Theander, Stefan R. Hansson, Morten A. Nielsen, Ali Salanti

**Affiliations:** 1 Centre for Medical Parasitology, Department of Immunology and Microbiology, University of Copenhagen and Copenhagen University Hospital, Denmark; 2 Vancouver Prostate Centre, Vancouver, BC, Canada; 3 Department of Biochemistry, Boston University School of Medicine, Boston, Massachusetts, United States of America; 4 Copenhagen Center for Glycomics and Department of Cellular and Molecular Medicine, University of Copenhagen, Denmark; 5 Michael Smith Genome Sciences Centre, British Columbia Cancer Agency, Vancouver, Canada; 6 Section of Environmental Health, Department of Public Health, University of Copenhagen, Copenhagen, Denmark; 7 Department of Obstetrics, Rigshospitalet, Faculty of Health and Medical Sciences, University of Copenhagen, Copenhagen, Denmark; 8 Division of Obstetrics and Gynecology, Department of Clinical Sciences, Lund University Hospital, Lund University, Lund, Sweden; Institute of Immunology and Infection Research, UNITED KINGDOM

## Abstract

During placental malaria, *Plasmodium falciparum* infected erythrocytes sequester in the placenta, causing health problems for both the mother and fetus. The specific adherence is mediated by the VAR2CSA protein, which binds to placental chondroitin sulfate (CS) on chondroitin sulfate proteoglycans (CSPGs) in the placental syncytium. However, the identity of the CSPG core protein and the cellular impact of the interaction have remain elusive. In this study we identified the specific CSPG core protein to which the CS is attached, and characterized its exact placental location. VAR2CSA pull-down experiments using placental extracts from whole placenta or syncytiotrophoblast microvillous cell membranes showed three distinct CSPGs available for VAR2CSA adherence. Further examination of these three CSPGs by immunofluorescence and proximity ligation assays showed that syndecan-1 is the main receptor for VAR2CSA mediated placental adherence. We further show that the commonly used placental choriocarcinoma cell line, BeWo, express a different set of proteoglycans than those present on placental syncytiotrophoblast and may not be the most biologically relevant model to study placental malaria. Syncytial fusion of the BeWo cells, triggered by forskolin treatment, caused an increased expression of placental CS-modified syndecan-1. In line with this, we show that rVAR2 binding to placental CS impairs syndecan-1-related Src signaling in forskolin treated BeWo cells, but not in untreated cells.

## Author Summary

*Plasmodium falciparum* is the most deadly malaria parasite, causing more than 500,000 deaths each year. The parasite infects the host’s red blood cells. In placental malaria infected red blood cells accumulate in placenta. The parasite protein VAR2CSA mediates this adherence, which causes complications for both mother and child. VAR2CSA binds a carbohydrate chain termed chondroitin sulfate (CS). CS is not a well-defined biochemical entity but constitute a family of oligosaccharides which each have unique sulfation patterns. The CS binding VAR2CSA is attached to proteoglycans expressed on the surface of placental cells. While much work has gone into understanding the nature of VAR2CSA and its interaction with placental CS, the protein to which the placental CS is attached is not known. To further the understanding of the molecular pathology of PM we characterized the CSPG receptor that the parasites adhere to by defining the exact proteoglycan that carries the placental CS. We further investigated the molecular and cellular consequences of VAR2CSA binding to the receptor. This work provides novel insights into the pathology of placental malaria and the nature of the parasite receptor. This may aid development of strategies to treat or prevent placental malaria.

## Introduction

Every year more than 500,000 people die from malaria. 90% of the mortality is caused by *Plasmodium falciparum*, the most deadly of the five *Plasmodium* species infecting humans [1, 2]. *P*. *falciparum* is especially virulent due to its unique capability of inserting members of the *Plasmodium falciparum* Erythrocyte Membrane Protein 1 (PfEMP1) protein family into the membrane of the infected erythrocyte. These proteins constitute an efficient survival mechanism by allowing the parasites to adhere to receptors in the vasculature of the host [3, 4], thereby avoiding immune system surveillance in the spleen [5–8]. In endemic areas, individuals develop immunity against malaria as they acquire antibodies capable of blocking parasite sequestration [9]. However, pregnant women are susceptible to infection, despite previously acquired immunity [5]. This has been associated with the expression of a serologically distinct PfEMP1 called VAR2CSA that enables specific sequestration in the placenta [10, 11].

During placental malaria, VAR2CSA expression allows the infected erythrocytes to adhere to chondroitin sulfate (CS) chains, a glycosaminoglycan (GAG) present on chondroitin sulfate proteoglycans (CSPGs) in the apical membrane of the placental syncytiotrophoblast [5, 11–13]. It has also been shown that infected erythrocytes sequester in the intervillous space, where the maternal blood circulates [14–16].

GAGs are linear polymers of repeated disaccharide units. In CS this unit consists of N-acetyl-D-Galactosamine (GalNAc) and hexuronic acid residues. While the base structure is simple, an immense heterogeneity is achieved by varying polymer length and modifications such as sulfation, which themselves vary along the saccharide chain [17]. Parasites expressing VAR2CSA accumulate preferentially in the placenta [14, 18]. This is despite the fact that CS is expressed throughout the vasculature of the human host [17]. This suggests that the placental CS is distinct from the CS expressed elsewhere and that the VAR2CSA protein has evolved to interact with this type of CS only. VAR2CSA expressing parasites preferentially adhere to CS carrying 4-O-sulfation of the GalNAc residues [5, 10]. CS is a common modification to a wide variety of proteins with different physiological functions, collectively termed chondroitin sulfate proteoglycans (CSPGs). These include CD44 [19], CSPG4 [20], decorin [21], versican [21], serglycin [22], syndecans [23], glypicans [21], and integrin α_5_β_1_ [24, 25]. While CS was identified as the receptor in placental malaria almost twenty years ago [5], the identity of the CSPG core protein has remained elusive. Without knowing the identity of the CSPG receptor we lack key molecular insights into the pathology of placental malaria. This impedes progress in understanding how parasite adhesion could activate or inactivate key signaling pathways in placental cells and how this relates to the detrimental effects of placental malaria on placental development and function.

In this study we sought to define the mechanism of parasite sequestration in the placenta. This included mapping the anatomic localization of the CPSG receptor, and identifying the specific CSPG core protein that supports parasite adhesion. For this we used recombinant VAR2CSA (rVAR2, [26, 27]) to pull-down CSPGs from placental homogenates and from syncytiotrophoblast microvillous membranes. This revealed that three distinct CSPGs were available for parasite adhesion in the placenta. Further investigation of these potential receptors by immunofluorescence (IF) and proximity ligation assay (PLA) on placental tissue and BeWo cells indicated syndecan-1 as a major CSPG receptor in placental malaria. This work has for the first time identified a specific CSPG receptor in placental malaria. This allows for a better understanding of the pathology and will aid in the development of tools to decrease the disease burden of placental malaria.

## Results

### Infected erythrocytes adhere to CSPGs on syncytiotrophoblast and in the intervillous space of the placenta

**Fig 1:**
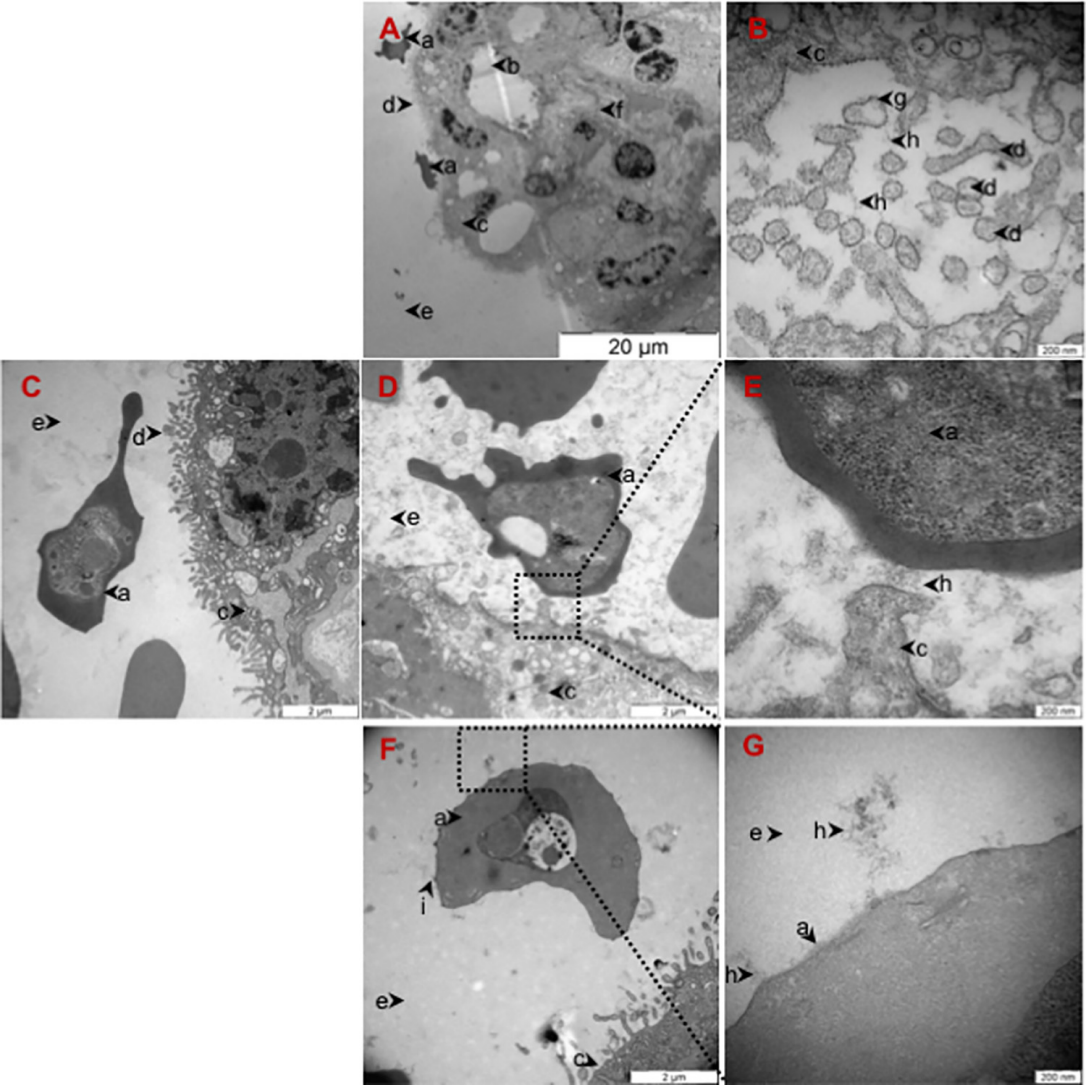
Infected Erythrocytes adhere to CS present on the syncytiotrophoblast and in the intervillous space in the placenta. (A) Transmission electron microscopy (TEM) of placental tissue glycosaminoglycans (GAGs) stained with 1% ruthenium red (RR) showing a fetal villus covered with syncytiotrophoblast. The surface of the syncytiotrophoblast is covered with microvilli. Two infected erythrocytes adhere to the apical membrane of the syncytiotrophoblast. Scale bar represents 20 μm. (B) TEM of placental tissue GAGs stained with 1% RR showing cross-sections of syncytiotrophoblast microvilli with the apical double layer plasma membrane surrounded by RR stained GAGs. Scale bar represents 200 nm. (C) TEM of control placental tissue perfused with VAR2CSA-expressing infected erythrocytes and not stained with RR. Scale bar represents 2 μm. (D) TEM of placental tissue perfused with VAR2CSA-expressing infected erythrocytes and stained with 1% RR showing the presence of GAGs where an infected erythrocyte is adhering to the syncytiotrophoblast membrane. Scale bar represents 2 μm.(E) TEM of higher magnification of the region outlined with a black square in (D) showing the presence of GAGs in between an infected erythrocyte and the syncytiotrophoblast. Scale bar represents 200 nm. (F) TEM of placental tissue stained with 1% RR showing an infected erythrocyte in the intervillous space close to the syncytiotrophoblast. Stained GAGs connected to knobs on an infected erythrocyte. Scale bar represents 2 μm. (G) TEM of higher magnification of the region outlined with a black square in (F) showing the presence of GAGs chains attached to knobs on an infected erythrocyte. Scale bar represents 200 nm.[a: infected erythrocyte; b: fetal capillary; c: syncytiotrophoblast; d: syncytiotrophoblast microvilli; e: intervillous space; f: villous stroma; g: double lipid layer; h: stained GAGs; i: knob].

**Fig 2:**
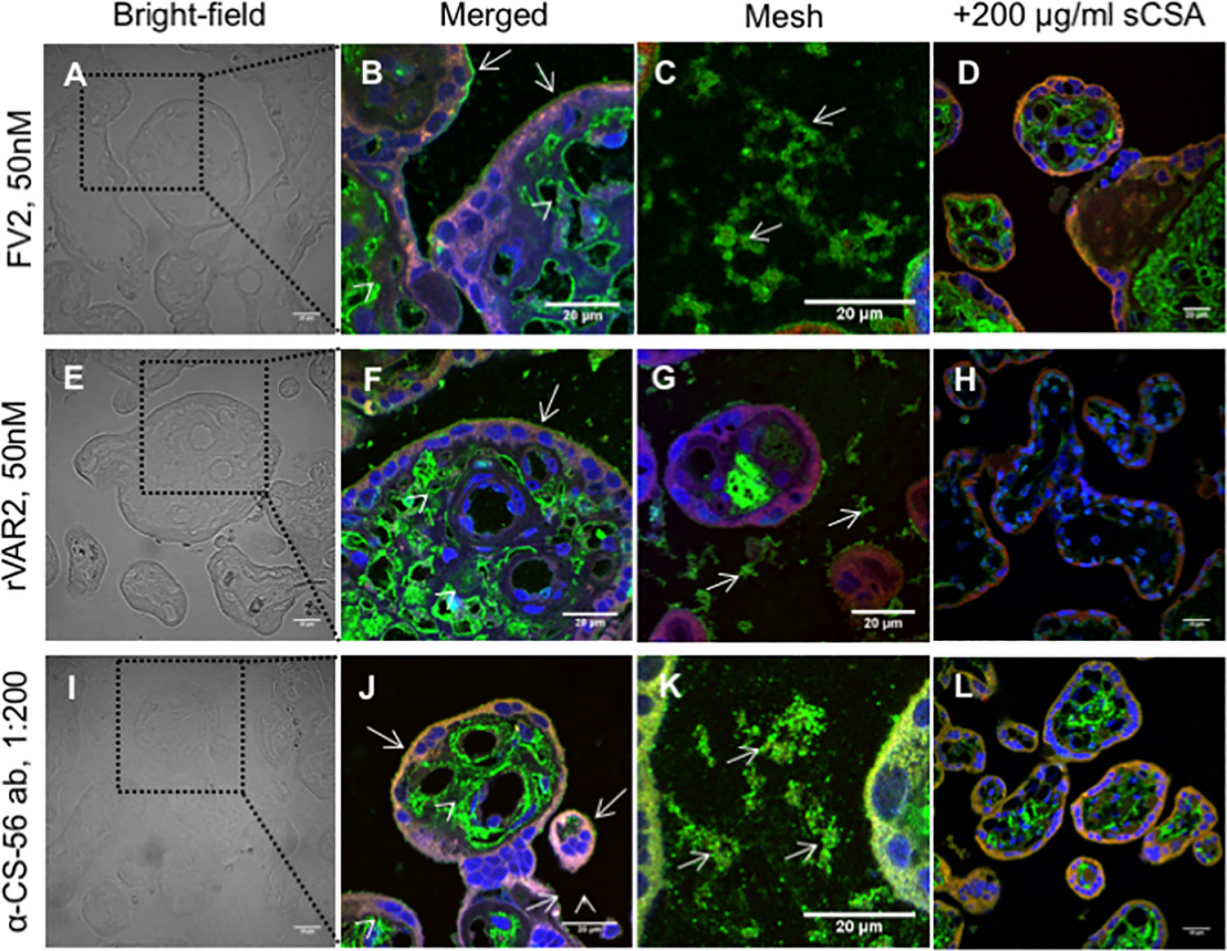
Placental chondroitin sulfate recognized by VAR2CSA is present on the syncytiotrophoblast and in the intervillous space of the placenta. Fresh placental tissue was perfused with VAR2CSA-expressing parasites before biopsies were collected and stained. A) Bright-field of placental tissue. The region delineated by a black dotted square is shown in (B). Scale bar represents 20 μm. (B) Full-length VAR2CSA (FV2) immunostaining show expression of placental CS in the villous stroma (arrow heads) and in the apical membrane of the syncytiotrophoblast (arrows). The image is a composite of three distinct channels: blue (nuclei), green (placental CS stained by FV2), and red (placenta auto-fluorescence). Scale bar represents 20 μm. (C) FV2 immunostaining showing the expression of placental CS in mesh-like structures (arrows). The image is a composite of three distinct channels: blue (nuclei), green (placental CS stained with FV2), and red (placenta auto-fluorescence). Scale bar represents 20 μm.(D) Addition of soluble CSA (sCSA) out-competes FV2 binding to placental tissue. The image is a composite of three distinct channels: blue (nuclei), green (placental CS stained with FV2), and red (placenta auto-fluorescence). Scale bar represents 20 μm. (E) Bright-field of placental tissue. The region delineated by a black dotted square is shown in (F). Scale bar represents 20 μm. (F) Recombinant VAR2CSA (rVAR2) immunostaining show the expression of placental CS in the villous stroma (arrow heads) and in the apical membrane of the syncytiotrophoblast (arrows). The image is a composite of three distinct channels: blue (nuclei), green (placental CS stained with rVAR2), and red (placenta auto-fluorescence). Scale bar represents 20 μm. (G) rVAR2 immunostaining show expression of placental CS in mesh-like structures (arrows). The image is a composite of three distinct channels: blue (nuclei), green (placental CS stained with rVAR2), and red (placenta auto-fluorescence). Scale bar represents 20 μm.(H) Addition of soluble CSA (sCSA) out-competes rVAR2 binding. The image is a composite of three distinct channels: blue (nuclei), green (placental CS stained with rVAR2), and red (placenta auto-fluorescence). Scale bar represents 20 μm. (I) Bright-field of placental tissue. A region delineated by a black dotted square is shown in (I). Scale bar represents 20 μm. (J) Anti-CS-56 antibody (α-CS-56 ab) immunostaining show the expression of CS in the villous stroma (arrow heads) and in the apical membrane of the syncytiotrophoblast (arrows). The image is a composite of three distinct channels: blue (nuclei), green (CS stained with α-CS-56 ab), and red (placenta auto-fluorescence). Scale bar represents 20 μm. (K) α-CS-56 ab immunostaining show the expression of CS in mesh-like structures (arrows). The image is a composite of three distinct channels: blue (nuclei), green (CS stained with α-CS-56 ab), and red (placenta auto-fluorescence). Scale bar represents 20 μm.m(L) Addition of soluble CSA (sCSA) out-competes α-CS-56 ab binding. The image is a composite of three distinct channels: blue (nuclei), green (CS stained with α-CS-56 ab), and red (placenta auto-fluorescence). Scale bar represents 20 μm.

To study the interaction between CS and placental-adhering parasites in detail we obtained placentas from healthy women at the time of delivery and perfused them with VAR2CSA-expressing infected erythrocytes [28]. Placental sections were stained with ruthenium red (RR) which stains polysaccharides, and investigated by transmission electron microscopy (TEM) to determine the ultra-structural localization of glycosaminoglycans (GAGs) (Fig 1A–1G). The results confirmed the presence of GAGs on the syncytiotrophoblast membrane, illustrated by the presence of electron-dense stained material in these regions (Fig 1A). We also observed stained GAGs on cross-sections of syncytiotrophoblast microvilli (Fig 1B). These structures were not visible in sections not stained by ruthenium red (Fig 1C). The ruthenium staining clearly demonstrated the presence of GAGs between infected erythrocytes and the syncytiotrophoblast membrane they were adhering to (Fig 1D and 1E). Furthermore, the knobs on the infected erythrocytes that were not in direct contact with the syncytium showed a single point of contact with long GAG chains in the intervillous space (Fig 1F and 1G). To determine if the GAG structures visualized by ruthenium red support VAR2CSA binding, we stained paraffin-embedded placental sections with full length recombinant VAR2CSA (FV2) and a truncated recombinant protein covering the CSA binding region of VAR2CSA (rVAR2) in immunofluorescence (IF) (Fig 2A–2H). FV2 and rVAR2 bound to CS present in the villi stroma, syncytiotrophoblast apical membrane and in the intervillous space (Fig 2A–2C for FV2 and Fig 2E–2G for rVAR2) in a pattern similar to that observed with ruthenium red. Addition of soluble bovine CSA (sCSA) in high concentrations outcompeted both FV2 and rVAR2 binding on the syncytiotrophoblast membrane and the intervillous space, confirming the CSA specificity of the VAR2CSA proteins (Fig 2D and 2H). Staining with the commercially available anti-CS (CS-56) antibody showed a similar pattern of CS expression, as the staining with the VAR2CSA reagents (Fig 2I–2L).

### Infected erythrocytes bind to a limited number of CSPGs in the placenta

Most studies characterizing the CSPG receptor for placental parasite adhesion have focused on the CS structure and not on the specific protein core to which the CS is attached, despite the fact that pathogenesis could be influenced by which proteoglycan interacts with the infected erythrocytes.

To identify the proteoglycan(s) involved in placental malaria, we performed pull-downs from placental tissue using rVAR2 and screened for placental CS-carrying CSPGs by mass spectrometry. To differentiate CSPGs involved in anchoring infected erythrocytes to the syncytiotrophoblast, we isolated the syncytiotrophoblast microvillous membrane using a Mg^2+^ precipitation protocol [29]. We identified four CSPGs highly expressed in the placenta: decorin (DCN), endorepellin (HSPG2), integrin beta 1 (ITGB1) and syndecan-1 (SDC1) (Table 1). In the pull-downs from crude placental tissue we only identified DCN and HSPG2. This likely reflects their high abundance in the ECM and basal membrane, respectively. Narrowing down the analyses to the syncytiotrophoblast membrane, we identified ITGB1 and SDC1 as the likely receptors for infected erythrocyte adhesion on the syncytiotrophoblast.

**Table 1:** Placental CSPGs, identified by mass spectrometry, from placenta extract or syncytiotrophoblast membrane vesicles binding rVAR2 in pull-downs experiments.

	Protein Name	Gene	Peptides Count	Seq. Coverage (%)	MS/MS Count	LFQ Ratio to Neg.#
*Whole placenta extract*	Decorin	*DCN*	13	40.7	77	110.49
	Endorepellin	*HSPG2*	18	5.0	20	225.15
*Syncytiotrophoblast Membrane Vesicles*	Syndecan-1	*SDC1*	3	15.2	27	∞§
	Integrin Beta-1	*ITGB1*	6	7.6	11	∞§

# label-free quantitation (LFQ) ratio to negative control

§ SDC1 and ITGB1 were not identified in control pull-down

**Fig 3:**
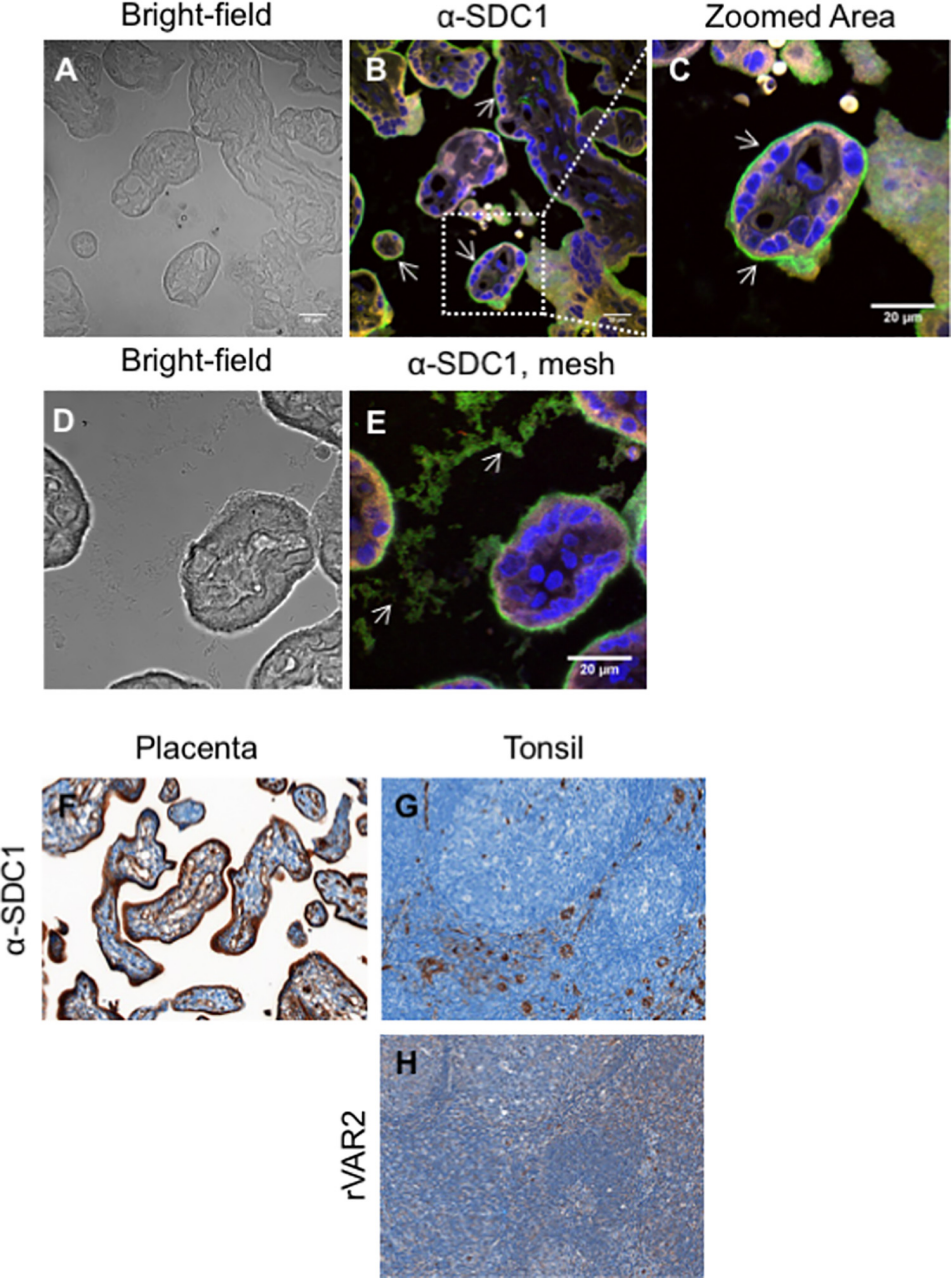
Expression of SDC1 in placental and tonsil tissue. (A) Bright-field of placental tissue perfused with VAR2CSA-expressing parasites showing infected erythrocytes adhering to the syncytiotrophoblast membrane. The same placenta region is shown stained in (B). (B) Immunostaining of paraffin-embedded placental tissue perfused with VAR2CSA-expressing parasites with an anti-syndecan-1 (SDC1) antibody showing SDC1 expression in the apical membrane of the syncytiotrophoblast (arrows). The image is a composite of three distinct channels: blue (nuclei), green (SDC1 staining), and red (placenta auto-fluorescence). Scale bar represents 20 μm. (C) Higher magnification of the region outlined with a white square in (B) showing more detail of placental SDC1 expression in the syncytiotrophoblast apical membrane (arrows). Scale bar represents 20μm. (D) Bright-field of placental tissue perfused with VAR2CSA-expressing parasites showing an infected erythrocyte adhering to the syncytiotrophoblast apical membrane and the presence of a mesh-like structure in the intervillous space. The same placenta region is shown stained in (E). (E) Immunostaining of paraffin-embedded placental tissue perfused with VAR2CSA-expressing parasites with an anti-syndecan-1 (SDC1) antibody showing the presence of SDC1 in the mesh-like structure present in the intervillous space (arrows). The image is a composite of three distinct channels: blue (nuclei), green (SDC1 staining), and red (placenta auto-fluorescence). Scale bar represents 20 μm. (F) Immunostaining of first-trimester placental tissue with an anti-syndecan-1 (SDC1) antibody showing the expression of SDC1 in the syncytiotrophoblast. Scale bar represents 20 μm. (G) Immunostaining of tonsil tissue with an anti-syndecan-1 (SDC1) antibody showing the expression of SDC1 in tonsil. Scale bar represents 20 μm. (H) Immunostaining of first-trimester placental tissue with rVAR2 showing the absence of the placental-type CS in the tonsil. Scale bar represents 20 μm.

To validate the importance of the identified proteoglycans we investigated the expression and localization of the CSPGs by immunofluorescence on paraffin-embedded placental sections perfused with infected erythrocytes (Fig 3A–3E and S1A–S1H Fig). The results showed that DCN is highly expressed in the villous stroma surrounding the fetal vessels, which are not accessible to the infected erythrocytes (S1A–S1C Fig). This is consistent with the pull-down of DCN from whole placenta extract and not from the syncytiotrophoblast membrane. ITGB1 was highly expressed on the apical side of the syncytiotrophoblast (S1D–S1F Fig) but a weak staining was also observed in the villi stroma surrounding fetal vessels (S1D–S1F Fig). *Bradbury et al*. has previously shown the presence of an amorphous structure in the intervillous space that could support parasite adhesion in the placenta [30]. Supporting such finding, we observed the presence of an amorphous structure in the intervillous space. ITGB1 was not present in this structure (S1G and S1H Fig). SDC1 was highly expressed on the apical side of syncytiotrophoblast (Fig 3A–3C) and was also present in the intervillous space (Fig 3D and 3E). In natural conditions, placental malaria infections occur throughout pregnancy. Therefore, we obtained first trimester (aborted) placental tissue. The data confirmed that SDC1 is also present on the syncytiotrophoblast in the first trimester placentas (Fig 3F).

The pull down experiment above indicated that VAR2CSA binds placental CS on syndecan-1. To investigate whether VAR2CSA also binds CS on syndecan-1 in other tissues we stained tonsil tissue with an anti-SDC1 antibody and with rVAR2. SDC1 was expressed in the tonsil tissue and was present in SDC1-rich regions (Fig 3G). rVAR2 did not stain the tonsil tissue, as described previously [31] (Fig 3H). This leads to the conclusion that VAR2CSA binds to CS expressed on syndecan-1 in the placenta, but not to syndecan-1 expressed by tonsil tissue. This also suggests that SDC1 is modified with a unique CS in the placenta, and that this CS is not expressed on syndecan-1 in tonsil tissue. The presence of a tissue specific placental CS modification on SDC1 would explain the tissue specificity of parasites expressing VAR2CSA and support SDC1 as a candidate receptor for binding of infected erythrocytes in placental malaria.

To confirm the specific presence of placental CS on the identified CSPGs we used proximity ligation assay (PLA) [32, 33] to test the co-localization between placental CS (rVAR2 stain) and the CSPG core protein in question. We co-stained paraffin-embedded placental sections, which had been perfused with VAR2CSA-expressing parasites, with specific monoclonal antibodies against the CSPG core proteins and the two VAR2CSA reagents, FV2 and rVAR2. The two components of the complex were targeted with secondary conjugated antibodies (PLA probes), which only when in close proximity (around 40nm) form circular DNA that is amplified and detected, producing a red dot upon a co-localization event. The results showed that DCN was highly substituted with placental CS but only present in the villous stroma (S2A–S2C Fig). ITGB1 did not carry placental CS on the apical side of the syncytiotrophoblast, as illustrated by the low number of signals observed (S2D–S2F Fig). This is in sharp contrast to the high expression of ITGB1 observed by IF in this membrane, and with the fact that ITGB1 was a major hit from the pull-downs performed on the isolated membrane. The results however, show that rVAR2 staining of placental CS co-localizes with ITGB1 on the basal side of the syncytiotrophoblast, facing the villous stroma. This suggests that ITGB1 does not always carry placental CS and likely reflects the so-called part-time CSPG nature of ITGB1 [24, 25]. In line with the absence of ITGB1 staining in the intervillous space, there was almost no co-localization signals at this location (S2G–S2H Fig).

**Fig 4:**
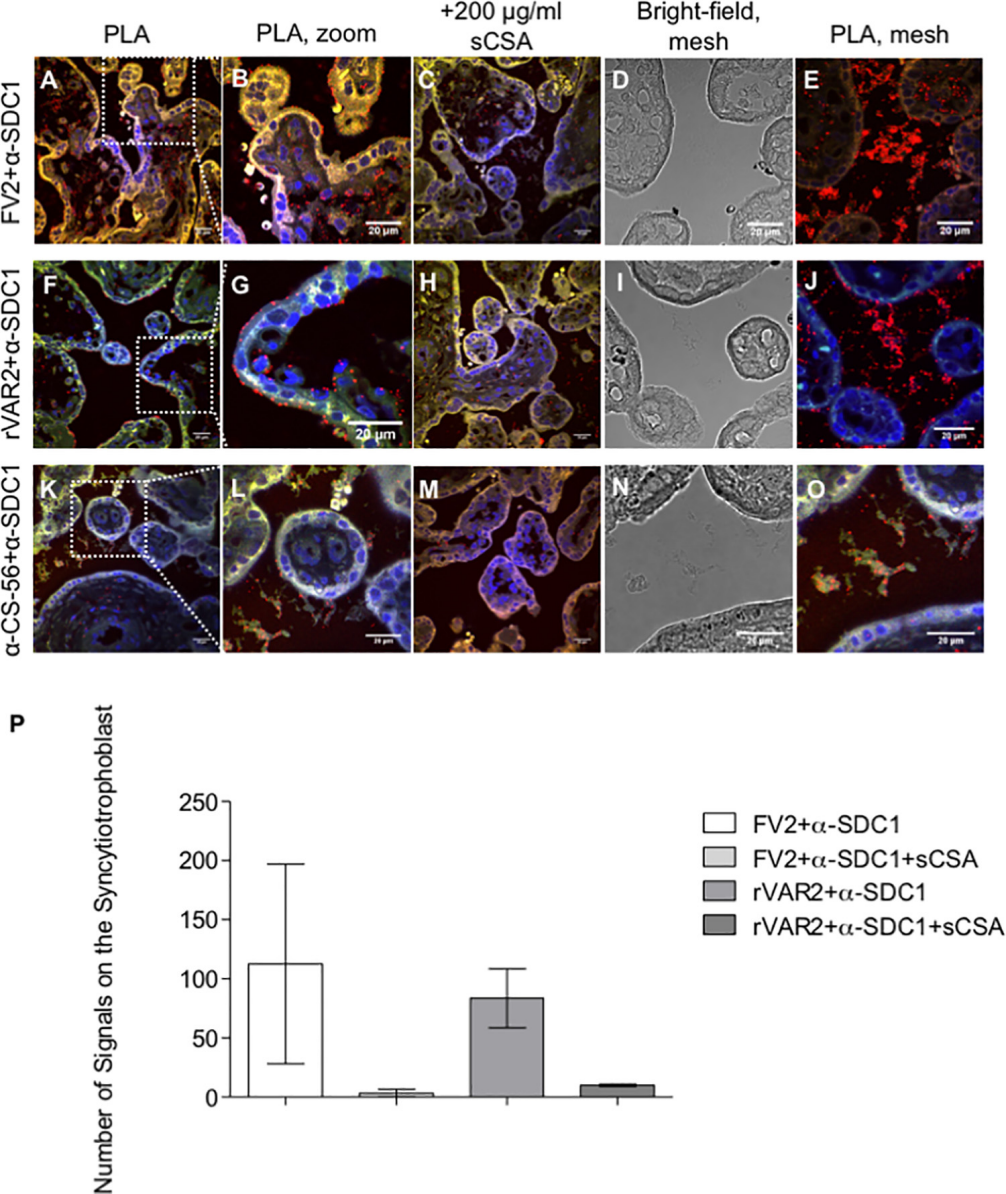
Co-localization between placental CS and SDC1 in third-trimester placental tissue. (A) Co-localization analysis by PLA between placental CS (FV2 stain) and syndecan-1 (SDC1) on paraffin-embedded placental tissue perfused with VAR2CSA-expressing parasites showing the Co-localization of SDC1 and placental CS chains in the syncytiotrophoblast and villous stroma (red dots). The image is a composite of three distinct channels: blue (nuclei), green (placenta auto-fluorescence), and red (co-localization between FV2 stain and SDC1). Scale bar represents 20 μm. (B) Higher magnification of the region outlined with a white square in (A) showing more detail of the co-localization between FV2 staining and SDC1 in the syncytiotrophoblast. Scale bar represents 20 μm. (C) Addition of soluble CSA (sCSA) confirms the specificity of FV2 by reducing the number of signals seen in the Co-localization between FV2 stain and SDC1. The image is a composite of three distinct channels: blue (nuclei), green (placenta auto-fluorescence), and red (co-localization between FV2 stain and SDC1). Scale bar represents 20 μm. (D) Bright-field visualization of placental tissue perfused with VAR2CSA-expressing parasites showing the presence of a mesh-like structure in the intervillous space. The same region is shown stained in (E). Scale bar represents 20 μm. (E) Co-localization of placental CS (FV2 stain) and syndecan-1 (SDC1) on mesh-like structure present in the intervillous space. The image is a composite of three distinct channels: blue (nuclei), green (placenta auto-fluorescence), and red (co-localization between FV2 stain and SDC1). Scale bar represents 20 μm. (F) Co-localization by PLA between placental CS (rVAR2 stain) and syndecan-1 (SDC1) on paraffin-embedded placental tissue perfused with VAR2CSA-expressing parasites showing the presence of placental CS carrying SDC1 on the apical side of the syncytiotrophoblast membrane (red dots). The image is a composite of three distinct channels: blue (nuclei), green (placenta auto-fluorescence), and red (co-localization between rVAR2 stain and SDC1). Scale bar represents 20 μm. (G) Higher magnification of the region outlined with a white square in (F) showing more detail of the co-localization between rVAR2 stain and SDC1 on the apical membrane of the syncytiotrophoblast. Picture shows an infected erythrocyte adhering in the syncytiotrophoblast close to co-localization events. Scale bar represents 20 μm. (H) Addition of soluble CSA (sCSA) confirms the specificity of rVAR2 by reducing the number of signals seen in the Co-localization between rVAR2 stain and SDC1. The image is a composite of three distinct channels: blue (nuclei), green (placenta auto-fluorescence), and red (co-localization between rVAR2 stain and SDC1). Scale bar represents 20 μm. (I) Bright-field visualization of placental tissue perfused with VAR2CSA-expressing parasites showing the presence of a mesh-like structure in the intervillous space. The same placenta region is shown stained in (J). (J) Co-localization by PLA between placental CS (rVAR2 stain) and SDC1 on paraffin-embedded placental tissue perfused with VAR2CSA-expressing parasites showing the presence of a high amount of placental CS carrying SDC1 in the mesh-like structure present in the intervillous space (red dots). The image is a composite of three distinct channels: blue (nuclei), green (placenta auto-fluorescence), and red (co-localization between rVAR2 stain and SDC1). Scale bar represents 20 μm. (K) Co-localization by PLA between CS (α-CS-56 antibody stain) and syndecan-1 (SDC1) on paraffin-embedded placental tissue perfused with VAR2CSA-expressing parasites showing the presence of CS on SDC1 in the syncytiotrophoblast (red dots). The image is a composite of three distinct channels: blue (nuclei), green (placenta auto-fluorescence), and red (co-localization between α-CS-56 antibody stain and SDC1). Scale bar represents 20 μm. (L) Higher magnification of the region outlined with a white square in (K) showing more detail of the co-localization between α-CS-56 antibody stain and SDC1 in the syncytiotrophoblast. Scale bar represents 20 μm. (M) Addition of soluble CSA (sCSA) does not decrease the detection of SDC1 with CS chains (detected with an α-CS-56 antibody) in the placental tissue perfused with VAR2CSA-expressing parasites. The image is a composite of three distinct channels: blue (nuclei), green (placenta auto-fluorescence), and red (co-localization between α-CS-56 antibody stain and SDC1). Scale bar represents 20 μm. (N) Bright-field visualization of placental tissue perfused with VAR2CSA-expressing parasites showing the presence of a mesh-like structure in the intervillous space. The same placenta region is shown stained in (O). (O) Co-localization by PLA between CS (α-CS-56 antibody stain) and SDC1 on paraffin-embedded placental tissue perfused with VAR2CSA-expressing parasites showing the presence of CS on SDC1 in the mesh-like structure present in the intervillous space (red dots). The image is a composite of three distinct channels: blue (nuclei), green (placenta auto-fluorescence), and red (co-localization between α-CS-56 antibody stain and SDC1). Scale bar represents 20 μm. (P) Quantification of the number of PLA signals representing the co-localization between SDC1 and CS (detected with FV2 and rVAR2) on the syncytiotrophoblast, in the presence or absence of sCSA. Signals were quantified manually. Values represent mean ± SEM.

Strong co-localization was detected with SDC1 on the apical membrane of the syncytiotrophoblast, suggesting that SDC1 carries placental CS in this region (Fig 4). In fact, several continuous PLA dots were present on the syncytiotrophoblast close to where infected erythrocytes had adhered. This was true for placental CS staining with both FV2 (Fig 4A and 4B) and rVAR2 (Fig 4F and 4G). Addition of sCSA decreased the number of co-localization signals considerably, confirming the CS specificity if the recombinant proteins (Fig 4C and 4H). Interestingly, we observed many PLA signals in the intervillous space with both FV2 (Fig 4D and 4E) and rVAR2 (Fig 4I and 4J), which correlates with the expression of placental CS and SDC1 in the IF. Some PLA signals were seen inside the villi, although the immunofluorescence results suggested that SDC1 is not present here. This is most likely due to differences in assay sensitivity. A quantification of the PLA signals present on the syncytiotrophoblast is shown in Fig 4P.

To further confirm the presence of CS on SDC1 we repeated the PLA setup using an anti-CS (CS-56) antibody to target the CS component. This showed some co-localization signals on the syncytiotrophoblast (Fig 4K and 4L) as well as in the mesh (Fig 4N and 4O). The addition of sCSA reduced the number of co-localization signals in the mesh, but had limited effect on antibody binding in the villi stroma (Fig 4M).

In combination, these results strongly suggest that SDC1 is the main receptor for the adherence of VAR2CSA-expressing infected erythrocytes in the placenta. Given the extremely low apical presence of placental CS on ITGB1, we tested if ITGB1 could function as a co-receptor in complex with SDC1 by examining the possible co-localization between ITGB1 and SDC1 by PLA. The results showed little or no co-localization between these two CSPGs (S2I–S2K Fig). Taken together, these results indicate that SDC1 is the primary receptor for adherence of VAR2CSA-expressing infected erythrocyte in healthy placentas collected at term.

### VAR2CSA-expressing parasites adhere to SDC1 extracted from human placenta

**Fig 5:**
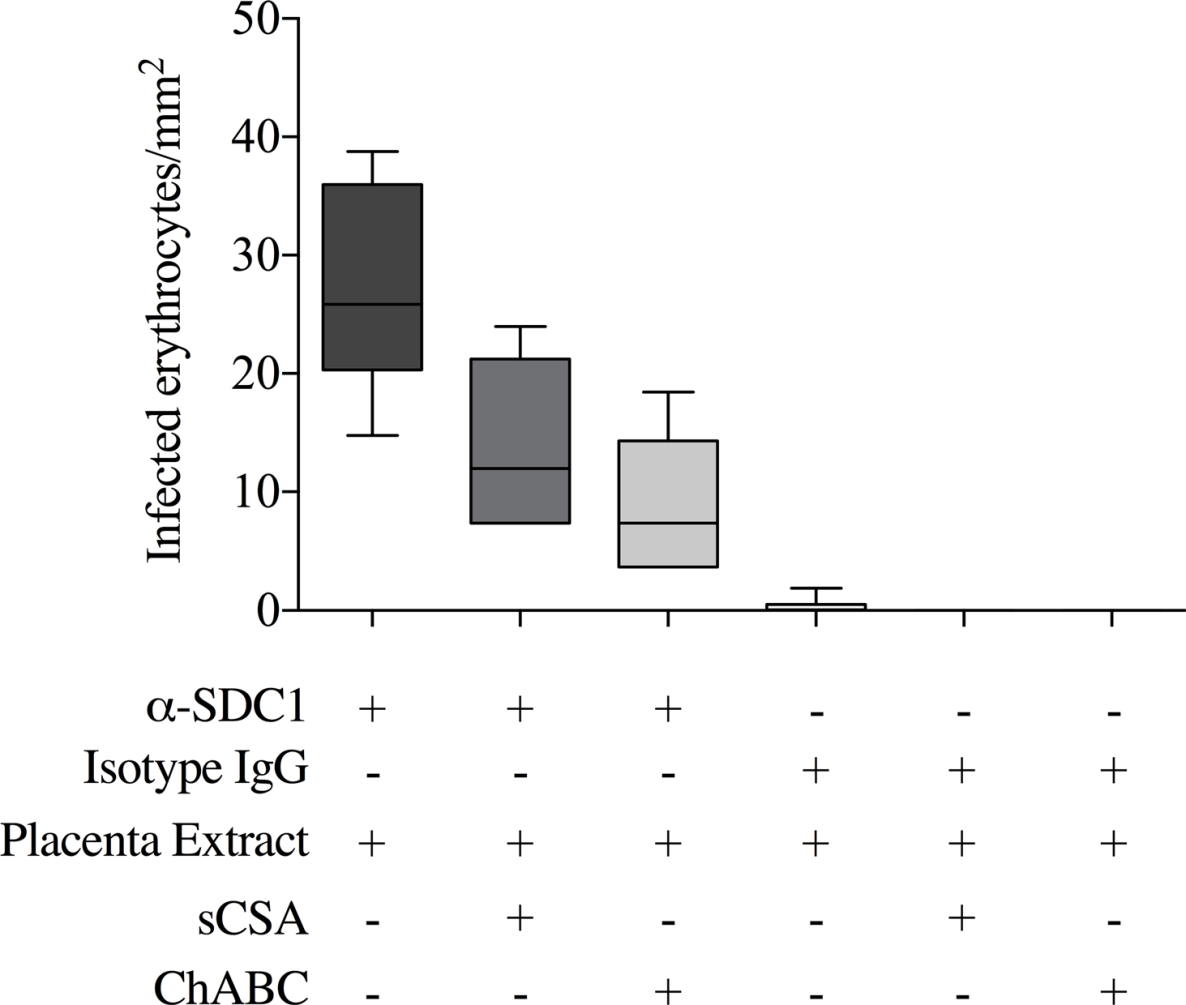
Binding of infected erythrocytes to placental SDC1. The figure shows the number of infected erythrocytes that bound (per mm^2^) to placental CSPG captured by an immobilized anti-SDC1 antibody or an isotype control IgG. For each setup, 2 replicates were run, and 3 representative pictures were taken for each of the replicates for quantification. The binding of the infected erythrocytes to SDC1 captured from the placental extract was confirmed in three independent experiments. sCSA denotes inhibition of binding using 400ug/mL soluble sigma CSA. ChABC denotes capture of CSPGs from placental extract pre-digested with Chondroitinase ABC.

Our results suggest that SDC1 is modified with a unique CS in the placenta, but that this CS is not present on SDC1 in other organs. To test if VAR2CSA-expressing parasites adhere to SDC1, we therefore investigated the interaction of the parasites with purified placental SDC1. To do this we tested parasite adhesion to placental SDC1 captured from a crude extraction of placental proteoglycans using an immobilized anti-SDC1 antibody. The parasites specifically adhered to the PG captured by the anti-SDC1 antibody, with no adhesion seen to the isotype IgG control (Fig 5). Furthermore, the interaction could be inhibited by soluble CSA and by ChABC treatment, illustrating the CS specificity of the interaction.

### BeWo cells as a model for placental CS and CSPG expression in the placenta

**Fig 6:**
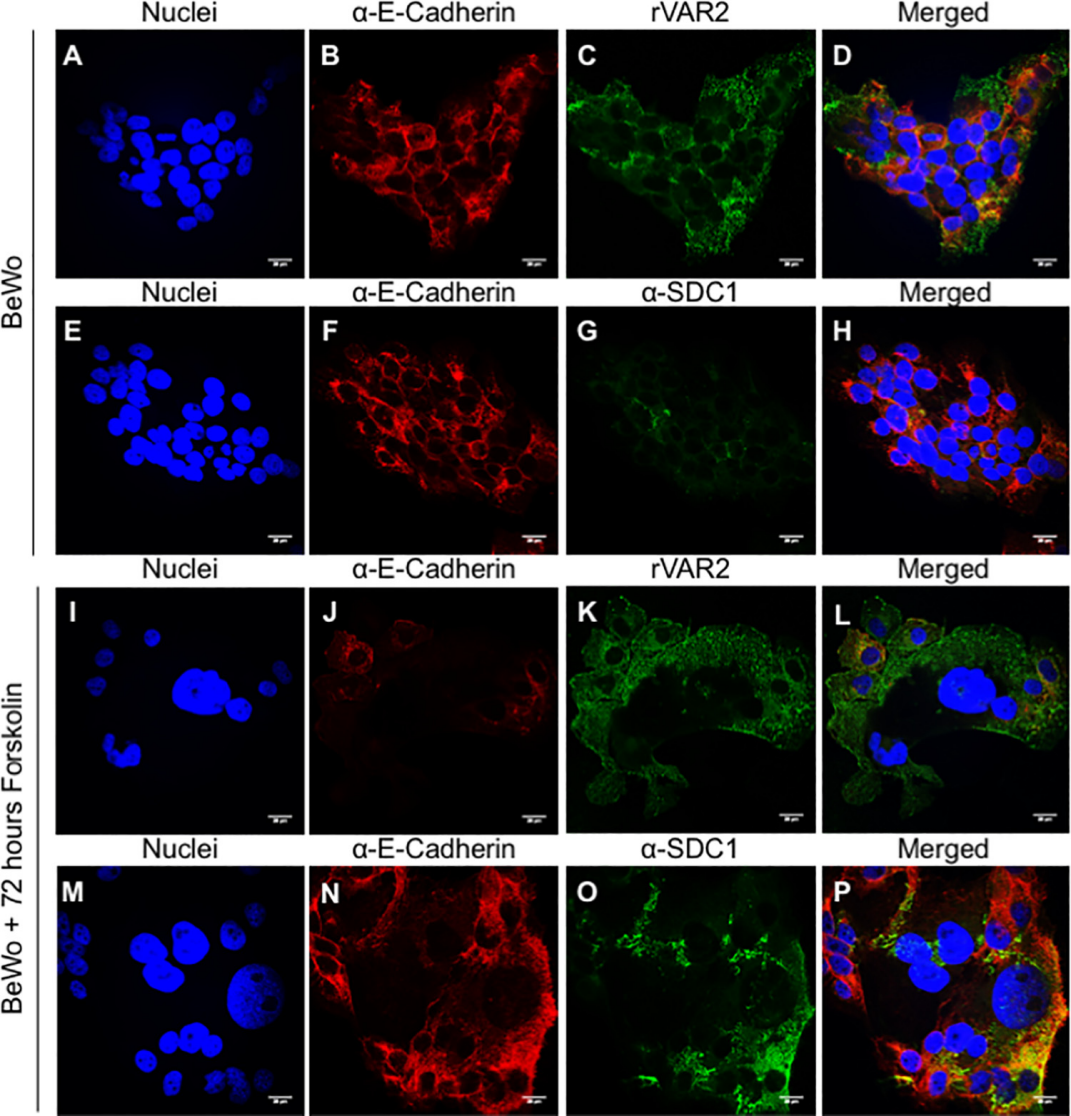
Expression of placental CS and SDC1 in BeWo cells before and after forskolin treatment by immunofluorescence. (A-D) Immunostaining of BeWo cells showing expression of E-cadherin (red) and placental CS at the cell surface (green). Cells are counterstained with DAPI (blue). Scale bar represents 20 μm. (E-H) Immunostaining of BeWo cells showing expression of E-cadherin (red) and syndecan-1 (SDC1) at the cell surface (green). Cells are counterstained with DAPI (blue). Scale bar represents 20 μm. (I-L) Immunostaining of BeWo cells showing expression of E-cadherin (red) and placental CS (green) at the cell surface after 72 hours of forskolin treatment. Cells are counterstained with DAPI (blue). Scale bar represents 20 μm. (M-P) Immunostaining of BeWo cells showing expression of E-cadherin (red) and syndecan-1 (SDC1) (green) at the cell surface after 72 hours of forskolin treatment. Cells are counterstained with DAPI (blue). Scale bar represents 20 μm.

The human trophoblastic BeWo cell line has been widely used as a model for the adherence of VAR2CSA-expressing parasites to placental CS [34–38]. BeWo cells consist mainly of cytotrophoblasts, cells that during placental development fuse and give rise to the syncytiotrophoblast. BeWo cells are incapable of differentiating into syncytiotrophoblast cells unless treated with cyclic AMP metabolism-related compounds like forskolin [39, 40]. To investigate the validity of the BeWo cell as a model for studying the cellular consequences of placental malaria infection we repeated the IF and PLA experiments performed with placental tissue on BeWo cells before and after forskolin treatment. To evaluate cellular fusion during the treatment, we stained the cells for E-cadherin, which allows the visualization of cell-cell contact points. The presence of placental CS on BeWo cells was confirmed by rVAR2 staining in IF. BeWo cells showed a punctuated surface staining for placental CS (Fig 6A–6D). However, the SDC1 (Fig 6E–6H) and DCN (S3A Fig) expression was weak. Contrary to this, ITGB1 expression was high (S3B Fig). 72 hours after forskolin treatment, expression of E-cadherin was markedly reduced compared to the non-treated control (Fig 6B and 6J). This indicated the formation of multinucleated cells and indicated that the cells had undergone a syncytialization process. The expression of placental CS was unchanged by the forskolin treatment (Fig 6K and 6L). However, SDC1 expression was markedly increased by the treatment (Fig 6O and 6P).

**Fig 7:**
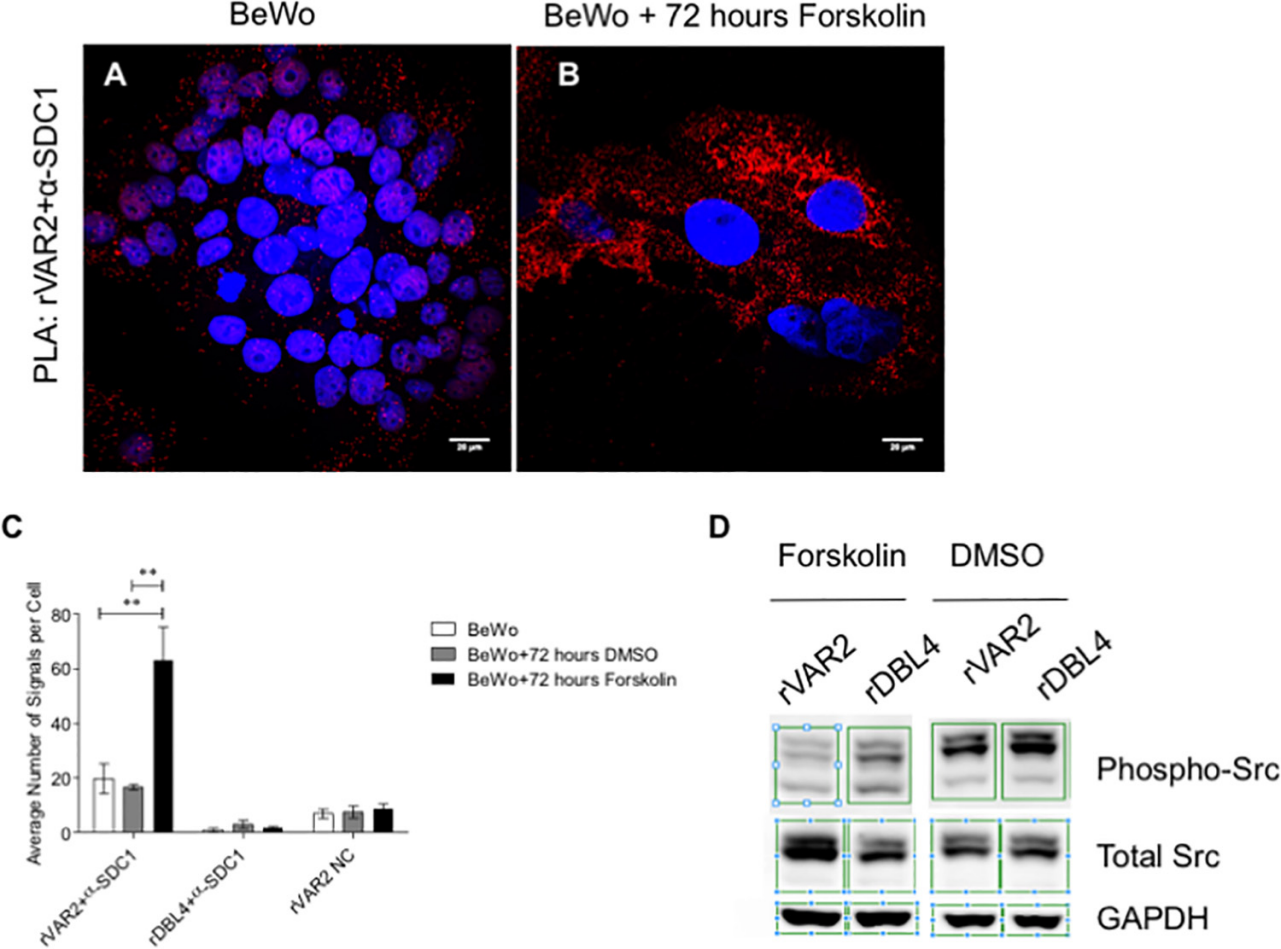
Co-localization between placental CS and SDC1 on BeWo cells before and after forskolin treatment, by PLA. **Signaling assay on BeWo cells before and after forskolin treatment.** (A) Co-localization by PLA between placental CS (rVAR2 stain) and syndecan-1 (SDC1) (red dots). Cells are counterstained with DAPI (blue). Scale bar represents 20 μm. (B) Co-localization by PLA between placental CS (rVAR2 stain) and syndecan-1(SDC1) shows the presence of high amounts of SDC1 with placental CS chains at the surface of BeWo cells after forskolin treatment for 72 hours (red dots). Cells are counterstained with DAPI (blue). Scale bar represents 20 μm. (C) Quantification of PLA signals for the co-localization between rVAR2 and SDC1 on BeWo cells, control cells incubated with DMSO, and cells after forskolin treatment. Values are presented as mean ± SEM. Two-way ANOVA test was used. Two asterisks (**) indicate a significant (P < 0.01) difference from both the non-treated BeWo cells, and cells treated with DMSO. (D) Signaling assay on BeWo cells treated with DMSO or Forskolin, and incubated for 24 hours with rVAR2 or the non-CS binding domain, rDBL4. Antibodies detecting GAPDH, phosphorylation of Src in Tyrosine 416 (p-Src) and total Src were used to assess the effect of rVAR2 binding to the Src-related signaling pathway.

Co-localization between rVAR2 staining and SDC1 in PLA showed that untreated BeWo cells express little SDC1 carrying placental CS, contrary to what was seen in placental tissue (Fig 7A). Interestingly, and consistent with the placenta data, we observed a statistically significant increase in the expression SDC1 carrying placental CS after forskolin treatment (Fig 7B and 7C). PLA results showed that DCN on BeWo cells does not carry placental CS (S3C Fig). Contrary to this, PLA showed many signals in the reaction between rVAR2 and the antibody against ITGB1 suggesting that this proteoglycan presents placental CS on the surface of BeWo cells (S3D Fig). Taken together, this shows that BeWo cells are not a good representative model for studying VAR2CSA mediated placental CSPG adhesion, but that forskolin induction of syncytialization leads to the expression of the placental receptor indicating that these treated cells provide a more suitable model for placental malaria research.

### rVAR2 binding to forskolin-treated BeWo cells impairs Syndecan-1 related intracellular signaling

Binding of infected erythrocytes to the placenta causes severe complications in pregnancy, including low birth weight and intra uterine growth retardation. Although a major reason for this is the unwarranted inflammation initiated by placental parasite sequestration, the VAR2CSA-SDC1 engagement could also impact on placental cell function. We thus incubated syncytialized BeWo cells with rVAR2 and observed its effects on the phosphorylation of Src, an intracellular signal protein working downstream of SDC1 [23, 41]. Analysis showed that binding of rVAR2 to these cells strongly inhibited the phosphorylation of Src (Fig 7D). The effect was only observed in forskolin treated BeWo cells, supporting the notion that the effect is mediated through the interaction between VAR2CSA and SDC1. The non-CS binding DBL4 VAR2CSA domain (rDBL4) did not affect Src signaling.

## Discussion

Placental malaria represents a unique phenomenon of organ specific sequestration of *P*. *falciparum*. In 1996 the placental receptor CSA was described by Fried and Duffy [5] and in 2003 the parasite ligand, VAR2CSA, was identified [10]. Immense efforts have since been invested in making an adhesion blocking vaccine [42]. However, this research has provided limited insight into the molecular and cellular determinants of placental sequestration. We investigated the mechanism underlying placental parasite adherence and identified the CSPG core proteins to which the placental CS is attached.

It has been shown that the minimal CS binding region of VAR2CSA is the DBL2X domain with the flanking interdomain regions [26]. Here we used this fragment (rVAR2) and the full-length VAR2CSA (FV2) to target placental CS in placental tissue. The binding was CSA specific, illustrated by the efficacy of soluble CSA (sCSA) in out-competing the VAR2CSA binding. The placental CS was present on the apical region of the syncytiotrophoblast and in the villous stroma. In order to relate VAR2CSA binding to the overall presence of CS, we compared the VAR2CSA staining with staining of matching placental tissue with a commercially available antibody against CS (CS-56). The pattern of staining with the antibody was similar to the staining seen with the two VAR2CSA reagents, FV2 and rVAR2. However, addition of sCSA only slightly reduced the binding of the CS-56 antibody to its target. This could be explained by the fact that the CS antibody targets both CSA and CSC [43].

The expression of placental CS on the maternal side of the placenta is a requirement for parasite adherence in the placenta. Previous studies have shown that some infected erythrocytes bind to the syncytiotrophoblast, but a large portion of the sequestered parasites are found in the intervillous space [14–16]. Bradbury *et al*. suggested that an amorphous structure in the intervillous space supports this parasite adherence [30]. Gowda *et al*. isolated CSPGs both from the intervillous space and placental tissue, and identified two distinct types of CSPGs that supported the adherence of infected erythrocytes in the placenta [15]. In accordance with these findings, we observed a matrix-type structure supporting VAR2CSA adherence, which was abundant in the intervillous space and accessible to infected erythrocytes. Further studies are necessary to characterize the nature of this structure and whether it plays a role in the pathogenesis of placental malaria.

To identify the placental CS-carrying CSPG supporting the adherence of infected erythrocytes in PM, we did pull-downs from isolated syncytiotrophoblast microvillous membrane using rVAR2. The subsequent proteomics screen revealed two major CSPG hits: ITGB1 and SDC1. ITGB1 is a transmembrane protein involved in cell-cell and cell-matrix interactions, cell migration and signaling. Previous studies have shown that ITGB1 expression is identical in normal placental tissue, in miscarriages, and in pregnancies complicated by preeclampsia [44, 45]. ITGB1expression was high on the apical side of the syncytiotrophoblast, but our co-localization PLA experiments indicated that ITGB1 does not to carry placental CS in this region. This most likely reflects the part-time CSPG status of ITGB1 [24, 25].

Syndecans are known components of the extracellular matrix and cell surface, and they can carry both heparan sulfate and chondroitin sulfate chains [46–48]. SDC1 has been shown to be crucial for placental implantation and angiogenesis [48] and is abundantly expressed on the apical side of the syncytiotrophoblast. Co-localization studies between CS (as shown by both FV2 and rVAR2 stain) and SDC1 revealed that this CSPG carries placental CS available for parasite adhesion. These signals could be out-competed with addition of sCSA. Some PLA signals were seen inside the villi, contrary to the fact that IF showed no expression of SDC1 here. This is most likely due to a difference in assay sensitivity. The IF showed continuous expression of SDC1 on the syncytiotrophoblast. The intermittent signals seen for the co-localization of SDC1 with FV2 and rVAR2 suggests that it does not always carry CS chains when on the apical side of the syncytiotrophoblast. To test the general CS substitution of SDC1 we tested co-localization between the staining with the commercially available CS-56 antibody and SDC1. This produced few signals compared to the staining with either FV2 or rVAR2, corresponding to the overall low staining of the placental tissue with CS-56 in IF. CS-56 recognizes a specific CSA/CSC epitope [43]. Thus, an explanation for the low staining is a lack of this specific target epitope in the placenta, or a low affinity of the antibody. To verify the placental CS specificity of VAR2CSA, we stained placental and tonsil tissue for SDC1 using an anti-SDC1 antibody and for placental CS using rVAR2. In this setup, tonsil represents a normal tissue with no placental CS expression, as described previously [31]. The staining showed that although SDC1 is present in tonsil it does not carry placental CS. This, together with the known expression of SDC1 in several human tissues [49], confirms our previous results and illustrates that the expression of SDC1 carrying placental CS is indeed specific to the placenta.

During normal pregnancy, SDC1 is shed from the syncytiotrophoblast into the maternal circulation [50, 51]. Interestingly, we found that both SDC1 and placental CS were specifically present in the mesh-like structures in the intervillous space. This was further confirmed by PLA showing the presence of SDC1 with placental CS chains in this region. This could explain the non-random accumulation of infected erythrocytes seen in the intervillous space of PM infected women [14–16] and it would be interesting to extract and directly analyze CSPGs from the intervillous space to confirm our results.

We have shown that SDC1 in the placenta carries placental CS targeted by rVAR2. To test if VAR2CSA-expressing infected erythrocytes interacted with placental SDC1 for adherence, we did a crude extraction of proteoglycans from placental tissue and pulled out SDC1 using an immobilized anti-SDC1 antibody. The infected erythrocytes adhered to the placental proteoglycan captured by the anti-SDC1 antibody and not by the isotype control. Furthermore, the interaction was CS specific as shown with sCSA inhibition and ChABC treatment. This validates the data collected with rVAR2 showing that SDC1 is the placental receptor for the placental malaria parasites and shows that the binding specificity is retained within the recombinant VAR2CSA proteins.

In an effort to identify the CSPG responsible for parasite adherence, Achur *et al*. did a series of extraction steps to purify CSPGs present in the intervillous space, on the cell surfaces, and in the fibrous tissue of the placenta [15]. They found several distinguishable CSPG fractions, but only two found in the intervillous space and one found in the cell surface extracts, efficiently supported infected erythrocyte adherence. The intervillous space fractions contained high molecular weight CSPGs which following chondroitinase ABC treatment revealed core proteins of 670kDa and 56kDa. In the cell surface extract they found a CSPG with a molecular weight of 250kDa and a core protein size in the 50-62kDa range [15, 52]. We identified placental CS-carrying syndecan-1 on the syncytiotrophoblast and in the intervillous space. Syndecan-1 is a CSPG with a core molecular weight around 20–40 kDa [53], and a total molecular weight ranging between 100–260 kDa [54], which lays in the range of the cell surface CSPG detected by Archur *et al*.. In a separate work, the SDC-1 shedded ectodomain was shown to have a molecular weight of 66kDa after heparinase and chondroitinase treatment [55], which could correspond to the smallest 56kDa CSPG found by Archur *et al*. in the intervillous space.

This work has identified SDC1 as an important binding partner for VAR2CSA-expressing parasites in the placenta. The experiments were primarily conducted on placental tissue from donor placentas collected at term from healthy women. Our investigation of first trimester placental tissue indicated that SDC1 is present and available for binding early in pregnancy, but we cannot rule out that other receptors may be available for adhesion in placentas during early gestation or become expressed as a result of acute or chronic inflammation induced by infection. Future investigations of placental biopsies from women with placental malaria are needed to clarify this.

The human choriocarcinoma BeWo cell line has been widely used as a model for the adherence of VAR2CSA-expressing parasites to placental CS [34–38], and as an *in vitro* model for studying the biochemical consequences of parasite binding in placental malaria [56–58]. Our results indicate that although the BeWo cells express placental CS, its expression of DCN and SDC1 does not correlate with that of the syncytiotrophoblast in the placenta. In fact, from the three CSPGs tested, only ITGB1 is highly expressed at the surface of the BeWo cells. DCN and SDC1 are expressed at low levels. Furthermore, contrary to what was observed in the placenta, ITGB1 was the only CSPG found to carry high amounts of placental CS by PLA. This suggests that the placental CS on untreated BeWo cells is attached to other CSPGs than SDC1, which appears to be the main binding partner for parasites in healthy placentas collected at term. BeWo cells consist mainly of cytotrophoblasts. In normal placental development cytotrophoblastic cells fuse and give rise to the syncytiotrophoblast. Treatment of BeWo cells with cyclic AMP metabolism-related compounds like forskolin induces BeWo syncytialization [39, 40]. To test if syncytialization of BeWo cells resulted in a similar CSPG expression pattern as seen on the placental syncytiotrophoblast, we investigated BeWo cells treated with forskolin. Forskolin treatment induced an increase in SDC1 expression. This is consistent with previous studies showing that BeWo cells have low SDC1 expression unless treated with forskolin [50]. The overall levels of placental CS were unchanged, consistent with studies showing that CS levels are unchanged when treating with forskolin [56]. Interestingly, forskolin treatment significantly increased the expression of placental CS-carrying SDC1. Taken together, these results indicate that BeWo cells are not an appropriate model for studying the down-stream cellular effects of infected erythrocytes adhesion, unless treated with compounds such as forskolin. Despite this, we believe that the BeWo cancer cell line can still be used as a cell line expressing placental CS for bio-panning of infected erythrocytes, since it does express other proteoglycans with placental type CS chains, though most cancer cell lines could be used for this purpose [31].

Src family protein tyrosine kinases (SFKs) are implicated in numerous signaling pathways, controlling many cellular functions such as cell growth, differentiation and migration [59, 60]. In the placenta, SFKs are involved in the differentiation of trophoblasts (the process in which cytotrophoblasts fuse and differentiate into syncytiotrophoblast) [61, 62]. Trophoblast differentiation impairment has been shown to lead to placental pathology such as pre-eclampsia [62]. Syndecan-1 is implicated in Src-related signaling and is thought to interact indirectly with Src [23, 41, 63]. Here we show that incubation of forskolin-treated BeWo cells with rVAR2 impairs Src-related signaling. This was not true for non-forskolin treated BeWo cells, suggesting that the effect seen on Src phosphorylation is through a rVAR2-mediated inhibition of placental CS substituted SDC1. Thus, parasite adhesion to syndecan-1 during early pregnancy could interfere with normal placental development and prevent the organ from reaching its full functional capacity. This in turn could help explain why malaria infections detected early in pregnancy can cause intra uterine growth retardation in the 3^rd^ trimester [64].

In this work we have characterized the structural basis for parasite binding in the placental. The major findings were: (1) VAR2CSA-expressing infected erythrocytes adhere in the human placenta through attachment to GAG chains present in the intervillous space and on the apical membrane of the syncytiotrophoblast; (2) SDC-1 is the primary CSPG binding partner for VAR2CSA in healthy human placentas collected at term; (3) BeWo cells do not share the complexity of the placental tissue unless treated with forskolin; (4) binding of VAR2CSA to forskolin treated BeWo cells impairs SDC1-related Src signaling.

This work provides valuable insight into the pathology of placental malaria and could pave the way for development of novel therapeutic compounds to treat or prevent this condition.

## Materials and Methods

### Placental tissue perfusions

Placental tissue for perfusion experiments were donated by healthy pregnant women delivering by caesarean section at term at Rigshospitalet University Hospital, Copenhagen, Denmark. The perfusion experiments have previously been described [28]. Briefly, the fetal and maternal circulation of a cotyledon was re-established in placentas obtained immediately after delivery and perfused with Krebs-Ringer buffer containing citrate 1:9 (fetal circulation) or RPMI (maternal circulation). Medium was then changed in both circulations to perfusion medium (RPMI 1640 with gentamicin and 1% red blood cells) and closed-loop perfusion was started. After stabilization phase the medium in the maternal circulation was changed to perfusion medium containing malaria infected erythrocytes. The placentas were perfused with FCR3 parasites selected for CSA binding and VAR2CSA expression on BeWo cells (ATCC CCL-98), as described before [34, 37]. One placenta was used for TEM experiments and one placenta for IF/PLA.

### Transmission electron microscopy (TEM)

The protocol used for preparing the tissue for TEM has been described before [28]. Small pieces (3x3x3 mm) of perfused tissue were fixed for 2 hours at RT in 1.5% paraformaldehyde (PFA, TAAB) and 1.5% glutaraldehyde (Sigma-Aldrich) in 0.1 M Sørensen buffer, pH 7.2. Tissue was primary stained during fixation with 0.5 or 1% ruthenium red (RR) or left unstained. After fixation, the tissue was washed in 0.1 M Sørensen buffer and stored O.N. at 4°C in 0.3 M Sørensen buffer. A secondary fixation was done by immersing the tissue in 1% osmium tetroxide. After this step, the tissue was dehydrated, embedded in Poly/Bed 812 (Polysciences), and cut with a Leica EM UC7 ultramicrotome into 50 nm sections. Lastly, sections were stained using 4% uranyl acetate and 1% lead citrate and observed under a Tecnai Spirit Biotwin (FEI Company) microscope.

### Isolation of syncytiotrophoblast microvillous membranes vesicles from human placenta

Placental tissue was obtained from pregnant women delivering at Rigshospitalet University Hospital, Copenhagen, Denmark. The protocol for isolation of microvillous membrane vesicles (MVM) was performed as described by Glazier *et al*. [29]. This protocol was repeated twice, and the isolated MVM from one experiment was used for subsequent pull-downs.

Briefly, the chorionic plate was removed, and the villous tissue was cut into small pieces and homogenized in Brown buffer (300 mM mannitol, 10 mM HEPES, 1 mM MgSO_4_, pH 7.4). This was followed by a Mg^2+^ precipitation by adding 10 mM MgCl_2_ while stirring on ice. After centrifugation at 2,300 g for 15 minutes at 4°C, the supernatant was centrifuged at 23,500 g for 40 minutes at 4°C. The obtained pellet was homogenized in Brown buffer and a second Mg^2+^ precipitation step followed. After this step, the homogenate was centrifuged at 2,300 g for 15 minutes at 4°C, and the obtained supernatant was centrifuged at 23,500 g for 40 minutes at 4°C. The final pellet was homogenized in intra-vesicular buffer (5 mM Tris, 5 mM HEPES, 290 mM sucrose, pH 7.4). The resulting homogenate was then forced to form vesicles by shear force by passing it through a 25G needle.

The purity and enrichment factor of the microvillous membranes (MVM), was assessed as previously described [65]. Alkaline Phosphatase (AP) was used as a marker for the MVM and an indicator of their purity. Its activity in the MVM was measured and normalized to that present in the initial placental homogenate. The enrichment factor of the MVM used for the experiments was 19.3.

### Pull-down assays

EBC lysis buffer (150 mM NaCl, 50 mM Tris-HCl, 2.5 mM MgCl_2_, 1 mM EDTA, 1% CHAPS and protease inhibitor cocktail (Roche)) was added to whole placenta tissue (from one placenta) and isolated syncytiotrophoblastic vesicles (from one placenta) to extract membrane proteins. The lysates were cleared by centrifugation. Biotinylated rVAR2 was added to the lysates and the mixture was incubated O.N. at 4°C. After that period, the rVAR2 and bound proteins were pulled-down using streptavidin coated dynabeads (MyOne C1, Invitrogen).

### Mass spectrometry analysis

After the pull-down assays, the lysates from the columns or protein bound to the dynabeads were dissolved in non-reducing LDS loading buffer (Invitrogen). The protein was reduced in 1 mM DTT and alkylated with 5.5 mM iodoacetamide. The samples were run 1 cm into Bis-Tris gels and stained with Commasie Blue. The gel was cut into cubes, washed and the proteins were in-gel digested with trypsin. The peptides resulted from the digestion were desalted using C18 Stage-tip [66], and analyzed by an Orbitrap Fusion Mass Spectrometer. Proteins were identified and quantified by MaxQuant using lable-free quantitation [67, 68], with a protein and peptide spectral match false discovery rate of 1%, and match between runs enabled. Samples were verified against pull-down by empty beads.

### Placental tissue and cell cultures for immunofluorescence (IF) and proximity ligation assay (PLA)

After perfusions, a full-thickness placental biopsy (from one perfused placenta) of approximately 1 cm^3^ was excised from the perfused cotyledon and fixed in 10% formalin at room temperature for further processing and paraffin-embedding. The specimens were cut to sections of 4 μm thickness. Before staining of the tissue, the paraffin was removed with xylene followed by rehydration of the sections in ethanol series. For antigen retrieval, the slides were heated up in a microwave in citrate buffer pH 6.0.

BeWo cells were acquired from the American Type Culture Collection (ATCC CCL-98). BeWo cells were allowed to grow O.N. at 37°C in coverslips in F-12 Ham Media (Sigma-Aldrich, N6658) supplemented with 10% FBS, 1% glutamine and 1% penstrep. For induction of cells syncytialization, cells were treated with 25 μM forskolin (Sigma-Aldrich, F6886) for 24, 48, and 72 hours. A 72 hours vehicle (DMSO) control was also run. Cells were then fixed in 4% PFA in PBS for 10 minutes at room temperature, followed by 3 washes with PBS. Fixed cultures were stored at 4°C until staining.

### Immunofluorescence assay (IF)

The rVAR2 proteins were used at 50 nM. The following primary antibodies were used for staining of placental tissue and BeWo cells: mouse monoclonal to Chondroitin Sulfate (1:200, clone CS-56, abcam, ab11570), rabbit polyclonal to decorin (1:100, Thermo Fisher Scientific, PA5-27370), rabbit monoclonal to E-cadherin (1:200, clone 24E10, Cell-Signaling, 3195) mouse monoclonal to integrin beta 1 (1:20, clone 4B7R, abcam, ab3167), mouse monoclonal antibody to syndecan 1 (used in BeWo stainings, 1:50, clone B-A38, abcam, ab34164) and rabbit polyclonal to syndecan 1 (used in placental tissue, 1:500, Sigma-Aldrich, HPA006185). The secondary antibodies used were: alexa fluor 555 anti-rabbit (1:500, Invitrogen, A31572), goat fluorescein anti-rabbit (1:500, Vector Laboratories, FI-1000), horse fluorescein anti-mouse (1:500, Vector Laboratories, FI-2000), and anti-V5-FITC antibody (1:500, Life Technologies, 46–0308) for detection of rVAR2.

After antigen retrieval or fixation (for placenta tissue and cell culture, respectively) specimens were blocked for 1 hour at RT in 1% BSA, 5% FBS in PBS. Specimens were incubated with the different primary antibodies or proteins diluted in 0.25% BSA in PBS for 1 hour at RT, followed by three washes of 5 minutes PBS. Lastly, the specimens were incubated with secondary antibodies for 45 minutes at RT in the dark and washed as described before. Duolink In Situ Mounting Medium with DAPI (DUO82040, Sigma-Aldrich) was applied and a glass coverslip added to the slide. Slides were stored at 4°C until further analysis using a confocal microscope. Negative controls were performed either by omitting the rVAR2 or primary antibody. For the CS staining, an additional control was performed by adding 200 μg/ml Sigma CSA (sCSA) to the protein/antibody solution. Each staining was performed at least twice.

### Proximity ligation assay (PLA)

The in situ PLA reagents were acquired from Sigma-Aldrich and the reaction was performed according to manufacturer’s instructions.

Following antigen retrieval or fixation (for placenta tissue and cell culture, respectively), specimens were stained with primary antibodies and rVAR2. Recombinant protein was used at 50 nM) and the following antibodies were used: mouse monoclonal to Chondroitin Sulfate (1:200, clone CS-56, abcam, ab11570), rabbit polyclonal to decorin (1:200 or 1:400 for BeWo cells and placental tissue, respectively, Thermo Fisher Scientific, PA5-27370), mouse monoclonal to integrin beta 1 (1:200, clone 4B7R, abcam, ab3167), mouse monoclonal antibody to syndecan-1 (used in BeWo stainings, 1:100, clone B-A38, abcam, ab34164) and rabbit polyclonal to syndecan-1 (used in placental tissue, 1:500, Sigma-Aldrich, HPA006185). Wash Buffer A (DUO82047) was used for the washes between each incubation unless stated otherwise. Following primary antibodies incubation, specimens were stained with anti-V5 (mouse or rabbit) antibody for recombinant protein detection, or goat anti-mouse IgG antibody for detection of the anti-CS antibody. After the incubation they were stained with Duolink In Situ PLA Probe Anti-Goat MINUS (DUO92006), Duolink In Situ PLA Probe Anti-Mouse MINUS (DUO92004) and Duolink In Situ PLA Probe Anti-Rabbit PLUS (DUO92002) diluted in Antibody Diluent (DUO82008). The specimens were then treated with the ligation solution, followed by incubation with the amplification solution. Both reagents for the ligation and amplification solutions were provided with the kit Duolink In Situ Detection Reagents Orange (DUO92007). After this incubation, cells were washed with Wash Buffer B (DUO82048). Slides were mounted using Duolink In Situ Mounting Medium with DAPI (DUO82040). Negative controls omitting one of the primary antibodies or protein were performed. Additionaly, a control was performed by adding 200 μg/ml soluble CSA (sCSA) to the protein/antibody solution. The images were analyzed using the free software BlobFinder (version 3.2.). Each staining was performed at least twice. For quantification of the PLA signals present in the syncytiotrophoblast, manual quantification was performed.

### Confocal microscopy

A Nikon C1 confocal microscope with a 60X oil objective was used for imaging the different specimens. A total of 5 representative pictures were taken per sample. Each image obtained is a composite of four distinct channels: blue (nuclei), bright-field, green (placental CS, DCN, ITGB1 and SDC1 staining for IF, and placenta auto-fluorescence for PLA) and red (placenta auto-fluorescence for IF, and PLA signals for PLA). The gain of the green and red lasers was set so that the auto-fluorescence from both channels was balanced. By doing so, we ensure that the observed signal is a positive signal, and not auto-fluorescence from the tissue.

### Immunohistochemistry (IHC)

Sectioned paraffin-embedded tissue samples were stained using the Ventana Discovery Platform. The slides were stained with 500 picomolar V5-tagged rVAR2, followed by detection with an anti-V5 antibody (1:700) and an anti-mouse-HRP antibody.

For SDC1 staining, antigen retrieval was performed using Tris-EDTA buffer for 32 minutes at 91°C using a Ventana Discovery Ultra autostainer. Following antigen retrieval, samples were incubated with primary antibody against SDC1 (1:200, rabbit monoclonal antibody, ab128936, abcam) for 60 minutes at 37°C, and lastly with universal secondary antibody for 32 minutes. Visualization was performed using Streptavidin-biotin peroxidase detection system and 3,3’-diaminobenzidine as chromogen.

### Crude extraction of placental proteoglycans

75 g of frozen non-perfused placental tissue (from one placenta) was minced and extensively washed with PBS. The tissue was mixed with 0.74mg/mL collagenase type IV (Life Technologies, Grand Island, NY) in 80 mL PBS containing Ca^2+^ and Mg^2^, incubated for 16 hours at 37°C and clarified by centrifugation at 3,000 g for 10 minutes. The supernatant was adjusted to 2 mM MgCl_2_ and 1% Triton X-100, treated with 1000 U Benzonase (Sigma-Aldrich), and incubated overnight at 37°C. The sample was clarified by centrifugation and mixed with DEAE resin. The resin was washed with wash buffer containing 20 mM NaOAc, pH 6.0, 100 mM NaCl, and the proteoglycans were eluted with elution buffer containing 20 mM NaOAc, pH 6.0, 1 M NaCl. The samples were dialysed against PBS using 30mL dialysis cassettes (Pierce).

### Parasite adhesion assay

The assay was performed as described by Fried *et al*. [42], with some small modifications. 22 spots in a Petri dish (Falcon 351029) were coated with 20 μL 0.05 mg/ml anti-SDC1 antibody (B-A38, ab34164, abcam) or 0.05 mg/ml isotype control antibody (anti-Androgen Receptor (AR) antibody, N-20, sc-816, Santa Cruz Biotechnology) and incubated at 37°C in a humid chamber for 2 h. After this, the spots were blocked with 3% BSA (Rockland) for 1 h at RT. The antibody-coated spots were then incubated with placental proteoglycan extract (that had been incubated with or without Chondroitinase ABC 0.5units/ml in PBS for 1 h at 37°C) for 1h at RT. FCR3 parasites selected on BeWo cells were MACS-purified for late stage infected erythrocytes. 5 x 10^6^ infected erythrocytes/ml in PBS with 2% FBS were pre-incubated with or without soluble CSA (Fluka) 400 μg/ml for 10 min at RT. 20 μL of the infected erythrocytes suspension was added to the spots and allowed to settle for 20 minutes at RT. Background binding was evaluated on BSA coated spots. Each sample was tested in duplicates. Unbound infected erythrocytes were washed off with PBS with 2% FBS on a rotation table three times and bound cells were fixed with 3% Glutaraldehyde (Fluka). Bound IE were visualized in a Nikon Eclipse TE2000-E and three images of each spot was taken using NIS-Elements F at 10 x magnification. Infected erythrocytes were counted in and converted to infected erythrocytes /mm^2^.

### Signaling assay

Sub-confluent Bewo cells were treated with 25 μM forskolin (Sigma-Aldrich, F6886) for 72 hours. Control cells were treated with DMSO. The treated cells were washed in PBS and serum starved in the presence of 100nM rVAR2 or rDBL4 control protein for 24 hours. Cells were then stimulated with 3% FBS for 1 hour. After this period, cells were placed on ice, washed 3 times in PBS and lysed in EBC lysis buffer with 0.5% NP40 and phosphatase and protease inhibitor cocktails (Roche). The protein concentration was determined and the samples were balanced against each other. The samples were run in western and probed for GAPDH (rabbit GAPDH monoclonal antibody, clone 14C10, #2118S, Cell Signaling) and p-Src (rabbit p-Src Family (Y416) monoclonal antibody, #2101S, Cell Signaling). For total protein determination, membranes were stripped and re-probed with GAPDH and Src antibody (rabbit Src antibody, #2108S, Cell Signaling). This experiment was repeated twice.

### Ethical considerations

Women participating in the perfusion study gave informed written consent before donation of their placenta. Placental tissue used for isolation of syncytiotrophoblast microvillous membrane vesicles was donated anonymously. Both studies were approved by the ethical review board in the Capital Region of Denmark (reference nr H-1-2012-103 and H-1-2014-088). Placental and tonsil tissue used for IHC was obtained from Vancouver General Hospital. All placental and tonsil tissue donors provided written informed consent.

## Supporting Information

S1 Fig Expression of Chondroitin Sulfate Proteoglycans (CSPGs) identified by MS in placental tissue. (A) Bright-field of placental tissue perfused with VAR2CSA-expressing parasites showing infected erythrocytes adhering to the apical syncytiotrophoblast membrane and in a mesh-like structure present in the intervillous space. The same placenta region is shown stained in (B-C). (B) Immunostaining of paraffin-embedded placental tissue perfused with VAR2CSA-expressing parasites with an anti-decorin (DCN) antibody showing decorin expression in the villous stroma and surrounding fetal capillaries. The image is a composite of three distinct channels: blue (nuclei), green (DCN staining), and red (placenta auto-fluorescence). Scale bar represents 20 μm. (C) Higher magnification of the region outlined with a white square in (B) showing more detail of placental DCN staining in the villous stroma and surrounding fetal capillaries. Scale bar represents 20 μm. (D) Bright-field of placental tissue perfused with VAR2CSA-expressing parasites showing infected erythrocytes adhering to the syncytiotrophoblast apical membrane. The same placenta region is shown stained in (E-F). (E) Immunostaining of paraffin-embedded placental tissue perfused with VAR2CSA-expressing parasites with an anti-integrin beta-1 (ITGB1) antibody showing ITGB1 expression in the apical membrane of the syncytiotrophoblast and in the villous stroma. The image is a composite of three distinct channels: blue (nuclei), green (ITGB1 staining), and red (placenta auto-fluorescence). Scale bar represents 20 μm. (F) Higher magnification of the region outlined with a white square in (E) showing more detail of placental ITGB1 staining in the syncytiotrophoblast apical membrane and villous stroma. Scale bar represents 20 μm. (G) Bright-field of placental tissue perfused with VAR2CSA-expressing parasites showing infected erythrocytes adhering in the syncytiotrophoblast apical membrane and the presence of a mesh-like structure in the intervillous space. The same placenta region is shown stained in (H). (H) Immunostaining of paraffin-embedded placental tissue perfused with VAR2CSA-expressing parasites with an anti-integrin beta-1 (ITGB1) antibody showing the absence of ITGB1 in the mesh-like structure present in the intervillous space. The image is a composite of three distinct channels: blue (nuclei), green (ITGB1 staining), and red (placenta auto-fluorescence). Scale bar represents 20 μm.
(TIF)

S2 Fig Co-localization in placental tissue between placental CS and the different CSPGs identified by mass spectrometry. (A) Bright-field of placental tissue perfused with VAR2CSA-expressing parasites. The same placenta region is shown stained in (B). (B) Co-localization by PLA between placental CS (rVAR2 stain) and decorin (DCN) on paraffin-embedded placental tissue perfused with VAR2CSA-expressing parasites showing the presence of DCN with placental CS chains in the villi stroma (red dots). The image is a composite of three distinct channels: blue (nuclei), green (placenta auto-fluorescence), and red (co-localization between rVAR2 and DCN). Scale bar represents 20 μm. (C) Higher magnification of the region outlined with a white square in (B) showing more detail of the co-localization between rVAR2 stain and DCN in the villous stroma surrounding fetal capillaries. Scale bar represents 20 μm. (D) Bright-field of placental tissue perfused with VAR2CSA-expressing parasites showing infected erythrocytes adhering in the syncytiotrophoblast apical membrane and in the intervillous space. The same placenta region is shown stained in (E). (E) Co-localization by PLA between placental CS (rVAR2 stain) and integrin beta-1 (ITGB1) on paraffin-embedded placental tissue perfused with VAR2CSA-expressing parasites showing the presence of ITGB1 with placental CS chains in the basal side of the syncytiotrophoblast membrane (red dots). The image is a composite of three distinct channels: blue (nuclei), green (placenta auto-fluorescence), and red (co-localization between rVAR2 and ITGB1). Scale bar represents 20 μm. (F) Higher magnification of the region outlined with a white square in (E) showing more detail of the co-localization between rVAR2 stain and ITGB1 in the basal membrane of the syncytiotrophoblast. Scale bar represents 20 μm. (G) Bright-field of placental tissue perfused with VAR2CSA-expressing parasites showing an infected erythrocyte adhering in the mesh-like structure in the intervillous space. The same placenta region is shown stained in (H). (H) Co-localization by PLA between placental CS (rVAR2 stain) and ITGB1 on paraffin-embedded placental tissue perfused with VAR2CSA-expressing parasites showing the presence of a low amount of ITGB1 with placental CS chains in the mesh-like structure present in the intervillous space (red dots). The image is a composite of three distinct channels: blue (nuclei), green (placenta auto-fluorescence), and red (co-localization between rVAR2 and ITGB1). Scale bar represents 20 μm. (I) Bright-field of placental tissue perfused with VAR2CSA-expressing parasites showing infected erythrocytes adhering in the syncytiotrophoblast apical membrane. The same placenta region is shown stained in (J). (J) Co-localization by PLA between ITGB1 and SDC1 on paraffin-embedded placental tissue perfused with VAR2CSA-expressing parasites showing that these two PGs do not co-localize on the syncytiotrophoblast membrane (red dots). The image is a composite of three distinct channels: blue (nuclei), green (placenta auto-fluorescence), and red (co-localization between ITGB1 and SDC1). Scale bar represents 20 μm. (K) Higher magnification of the region outlined with a white square in (J) showing more detail of the co-localization between ITGB1 and SDC1. Scale bar represents 20 μm.
(TIF)

S3 Fig Expression of DCN and ITGB1 by IF and co-localization between placental CS and the different CSPGs on BeWo cells by PLA. (A) Immunostaining of decorin (DCN green) in BeWo cells. Cells are counterstained with DAPI (blue). Scale bar represents 20 μm. (B) Immunostaining of integrin beta-1 (ITGB1, green) in BeWo cells. Cells are counterstained with DAPI (blue). Scale bar represents 20 μm. (C) Co-localization by PLA between placental CS (rVAR2 stain) and decorin (DCN) shows the presence of low amounts of DCN with placental CS chains at the surface of BeWo cells (red dots). Cells are counterstained with DAPI (blue). Scale bar represents 20 μm. (D) Co-localization by PLA between placental CS (rVAR2 stain) and integrin beta-1 (ITGB1) shows the presence of high amounts of ITGB1 with placental CS chains at the surface of BeWo cells (red dots). Cells are counterstained with DAPI (blue). Scale bar represents 20 μm.
(TIF)
