# Supplementary figures and images for: Placental Sequestration of Plasmodium falciparum Malaria Parasites Is Mediated by the Interaction Between VAR2CSA and Chondroitin Sulfate A on Syndecan-1
Source: PLoS Pathog. 2016 Aug 24;12(8):e1005831. doi: 10.1371/journal.ppat.1005831 (PMC4996535; doi:10.1371/journal.ppat.1005831)

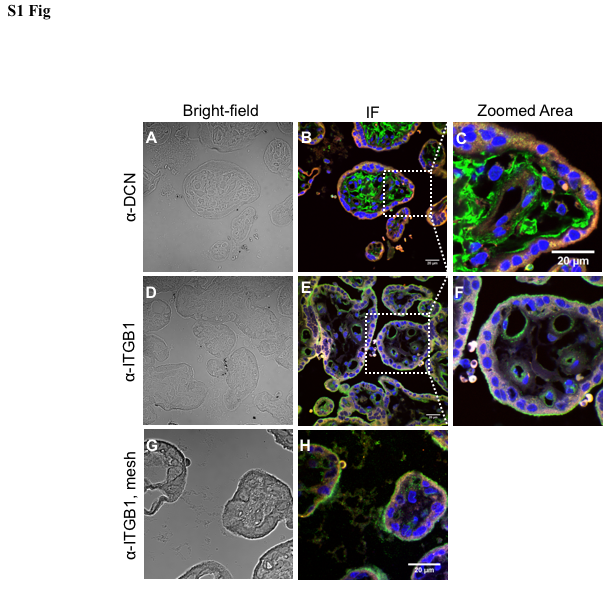

Supplement: S1 Fig — (A) Bright-field of placental tissue perfused with VAR2CSA-expressing parasites showing infected erythrocytes adhering to the apical syncytiotrophoblast membrane and in a mesh-like structure present in the intervillous space. The same placenta region is shown stained in (B-C). (B) Immunostaining of paraffin-embedded placental tissue perfused with VAR2CSA-expressing parasites with an anti-decorin (DCN) antibody showing decorin expression in the villous stroma and surrounding fetal capillaries. The image is a composite of three distinct channels: blue (nuclei), green (DCN staining), and red (placenta auto-fluorescence). Scale bar represents 20 μm. (C) Higher magnification of the region outlined with a white square in (B) showing more detail of placental DCN staining in the villous stroma and surrounding fetal capillaries. Scale bar represents 20 μm. (D) Bright-field of placental tissue perfused with VAR2CSA-expressing parasites showing infected erythrocytes adhering to the syncytiotrophoblast apical membrane. The same placenta region is shown stained in (E-F). (E) Immunostaining of paraffin-embedded placental tissue perfused with VAR2CSA-expressing parasites with an anti-integrin beta-1 (ITGB1) antibody showing ITGB1 expression in the apical membrane of the syncytiotrophoblast and in the villous stroma. The image is a composite of three distinct channels: blue (nuclei), green (ITGB1 staining), and red (placenta auto-fluorescence). Scale bar represents 20 μm. (F) Higher magnification of the region outlined with a white square in (E) showing more detail of placental ITGB1 staining in the syncytiotrophoblast apical membrane and villous stroma. Scale bar represents 20 μm. (G) Bright-field of placental tissue perfused with VAR2CSA-expressing parasites showing infected erythrocytes adhering in the syncytiotrophoblast apical membrane and the presence of a mesh-like structure in the intervillous space. The same placenta region is shown stained in (H). (H) Immunostaining of pa [file ppat.1005831.s001.tif]

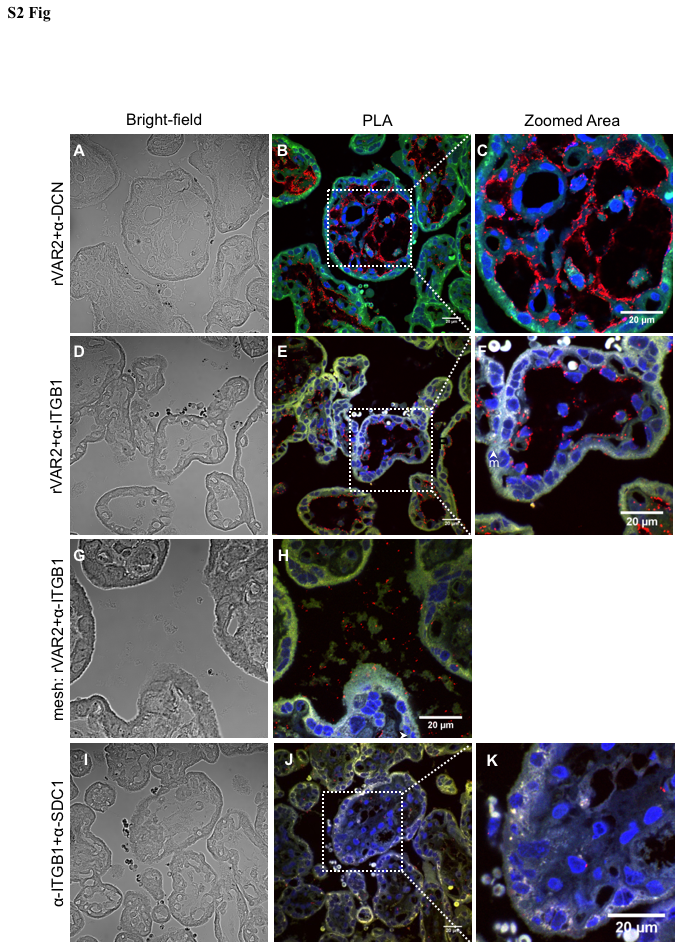

Supplement: S2 Fig — (A) Bright-field of placental tissue perfused with VAR2CSA-expressing parasites. The same placenta region is shown stained in (B). (B) Co-localization by PLA between placental CS (rVAR2 stain) and decorin (DCN) on paraffin-embedded placental tissue perfused with VAR2CSA-expressing parasites showing the presence of DCN with placental CS chains in the villi stroma (red dots). The image is a composite of three distinct channels: blue (nuclei), green (placenta auto-fluorescence), and red (co-localization between rVAR2 and DCN). Scale bar represents 20 μm. (C) Higher magnification of the region outlined with a white square in (B) showing more detail of the co-localization between rVAR2 stain and DCN in the villous stroma surrounding fetal capillaries. Scale bar represents 20 μm. (D) Bright-field of placental tissue perfused with VAR2CSA-expressing parasites showing infected erythrocytes adhering in the syncytiotrophoblast apical membrane and in the intervillous space. The same placenta region is shown stained in (E). (E) Co-localization by PLA between placental CS (rVAR2 stain) and integrin beta-1 (ITGB1) on paraffin-embedded placental tissue perfused with VAR2CSA-expressing parasites showing the presence of ITGB1 with placental CS chains in the basal side of the syncytiotrophoblast membrane (red dots). The image is a composite of three distinct channels: blue (nuclei), green (placenta auto-fluorescence), and red (co-localization between rVAR2 and ITGB1). Scale bar represents 20 μm. (F) Higher magnification of the region outlined with a white square in (E) showing more detail of the co-localization between rVAR2 stain and ITGB1 in the basal membrane of the syncytiotrophoblast. Scale bar represents 20 μm. (G) Bright-field of placental tissue perfused with VAR2CSA-expressing parasites showing an infected erythrocyte adhering in the mesh-like structure in the intervillous space. The same placenta region is shown stained in (H). (H) Co-localization by PLA between placental C [file ppat.1005831.s002.tif]

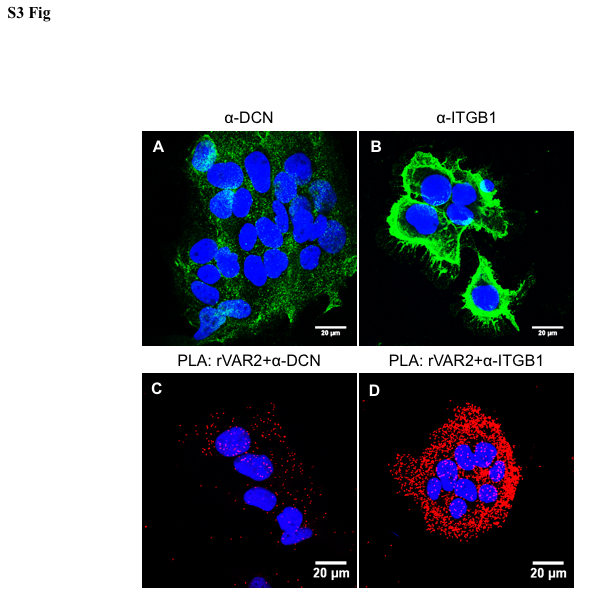

Supplement: S3 Fig — (A) Immunostaining of decorin (DCN green) in BeWo cells. Cells are counterstained with DAPI (blue). Scale bar represents 20 μm. (B) Immunostaining of integrin beta-1 (ITGB1, green) in BeWo cells. Cells are counterstained with DAPI (blue). Scale bar represents 20 μm. (C) Co-localization by PLA between placental CS (rVAR2 stain) and decorin (DCN) shows the presence of low amounts of DCN with placental CS chains at the surface of BeWo cells (red dots). Cells are counterstained with DAPI (blue). Scale bar represents 20 μm. (D) Co-localization by PLA between placental CS (rVAR2 stain) and integrin beta-1 (ITGB1) shows the presence of high amounts of ITGB1 with placental CS chains at the surface of BeWo cells (red dots). Cells are counterstained with DAPI (blue). Scale bar represents 20 μm. (TIF) [file ppat.1005831.s003.tif]
